# Action recognition in medical environments for robotic assistance

**DOI:** 10.1007/s11548-025-03551-6

**Published:** 2025-11-24

**Authors:** Sonja Stabenow, Lars Wagner, Alois Knoll, Klaus Bengler, Dirk Wilhelm

**Affiliations:** 1 Technical University of Munich, School of Medicine and Health, TUM University Hospital Rechts der Isar, Research Group MITI, Munich, Germany; 2 Technical University of Munich, TUM School of Computation, Information and Technology, Chair of Robotics, Artificial Intelligence and Real-Time Systems, Munich, Germany; 3 Chair of Ergonomics, TUM School of Engineering and Design, Technical University of Munich, Munich, Germany; 4 Department of Surgery, TUM School of Medicine and Health, TUM University Hospital Rechts der Isar, Technical University of Munich, Munich, Germany

**Keywords:** Human action recognition, Surgery, Patient ward, Robot, Healthcare, Human-robot interaction

## Abstract

**Purpose:**

Teamwork is fundamental to medical practice and relies on seamless collaboration among professionals with different tasks. Integrating robotic systems into this environment demands smooth interactions. Human action recognition, which infers a person’s state without explicit input, can support this. We focus on handovers between medical staff, using the actions as implicit cues for robotic assistance to replace the giving party in such scenarios.

**Methods:**

Skeletal information processed with differing machine learning algorithms makes it possible to derive actions out of sequential image data. Transferred to the medical context, we aim to infer actions defined for each situation in two datasets, a surgery in the operating room and a care intervention in the patient ward, depicting a handover between staff. We aim to abstract movement patterns across individuals through skeletal representation, leveraging the spatiotemporal information of medical handovers to enable future robotic systems to interact based on implicit cues.

**Results:**

We report an *F*1 score of $0.736 \pm 0.045$ for the OR dataset with ST-GCN and an *F*1 score of $0.941 \pm 0.009$ for the Ward dataset with the SkateFormer human action recognition. The defined actions showed distinction in the confusion matrix with limitations on actions with a rapid transition like approach and reach as well as the handover actions in the OR.

**Conclusion:**

The handover phases in two medical contexts, a minimally invasive surgery and a wound dressing on the patient station, are recognized with the proposed framework. This lays a first step for the integration of robotic assistance in the handover of medical material or instruments.

## Introduction

In healthcare, the integration of assistive technology is increasing to meet the demands of new standards of care and a growing shortage of skilled professionals. According to medical staff, the primary goals of introducing assistive systems are to alleviate workload and automate physical or time-consuming tasks [1].

In the *operating room* (OR), one of the tasks that has been explored for robotic assistance is the work of sterile nurses providing instruments for the surgeon [2, 3]. During surgery, nurses play an important role by responding to both explicit requests and implicit cues. Nurses rely on their knowledge of surgical procedures and workflow patterns to know when the surgeon needs what kind of instrument. As robots are increasingly integrated into the OR in research applications to address nursing shortages, they are expected to replicate the fluid, context-aware actions of nurses. To enable this interaction, we will focus on the recognition of the handover to map when an interaction is expected implicitly.

In the *patient ward* (Ward), robotic systems are starting to be introduced for fetch and bring tasks [4]. Adapting robotic systems to work with and cooperate with medical staff is the key to bringing adoption forward [1]. The situation of patient care needs special focus as similarly to the OR the focus of the medical professional is on the patient. Therefore, we focus on the fluent handover between robotic assistive systems and healthcare professionals during patient interventions. In both settings, the operating room and the patient ward, the transfer of items can be identified as a sequence of interest for enhancing teamwork with robotic systems.

As proposed by Li et al. [5], proactive human–robot collaboration is the step toward shared work for a common goal. Anticipating human actions may enhance productivity and trust, making dynamic adaptation and accurate prediction important milestones for seamless human–robot integration. To achieve this, the authors suggest that spatiotemporal cooperation prediction must be accomplished. Therefore, the focus is not limited to action prediction at each point in time but also on extracting temporal knowledge over time.

Similarly, Su et al. [6] postulate that the key challenge for developing surgical robotics is human activity acquisition. The vision painted by the authors shows an integrated OR where robotic systems and medical staff work hand in hand. The expectation in the OR is that all involved staff have a level of common knowledge to react to each other and show regard. Cooperation in this does not become a feature but a necessity for the successful integration of robotic systems. Motivated by this, we will apply human action recognition techniques on two datasets, the Ward dataset and the OR dataset with a focus on handover actions.

*Human action recognition* (HAR) identifies the state and or dynamic activities of a person or group derived from sensor input like RGB images. The skeleton-based approach is a common abstraction for a more robust recognition with lower computational cost. The human pose is described by several keypoints, usually placed at joints or significant landmarks like eyes or nose, representing the body. The connection of the points by vectors or bones results in a human-shaped representation, which is often called a skeleton. Our work will base the enumeration and representation on the COCO dataset annotation [7]. HAR with pose recognition is a two-step recognition process that leverages computer vision for the skeleton and machine learning techniques to transfer the pose data into action classes.

Human action recognition is a complex computation task trained and tested often on video datasets for short- or long-sequence recognition. Gathering data is a base step for HAR, and publicly available data sets often face the difficulty of diversity of the subjects or diversity of scenes [8]. As of our knowledge, there have not been medical scenarios investigated for human action recognition for robotic assistance.

Sarkar et al. [8] suggest that while high benchmark scores provide insight into model performance, their transferability may be limited, as the significance of actions varies by context and the intended purpose. We aim to explore the effectiveness of common HAR techniques on the presented scenes.

**Fig. 1:**
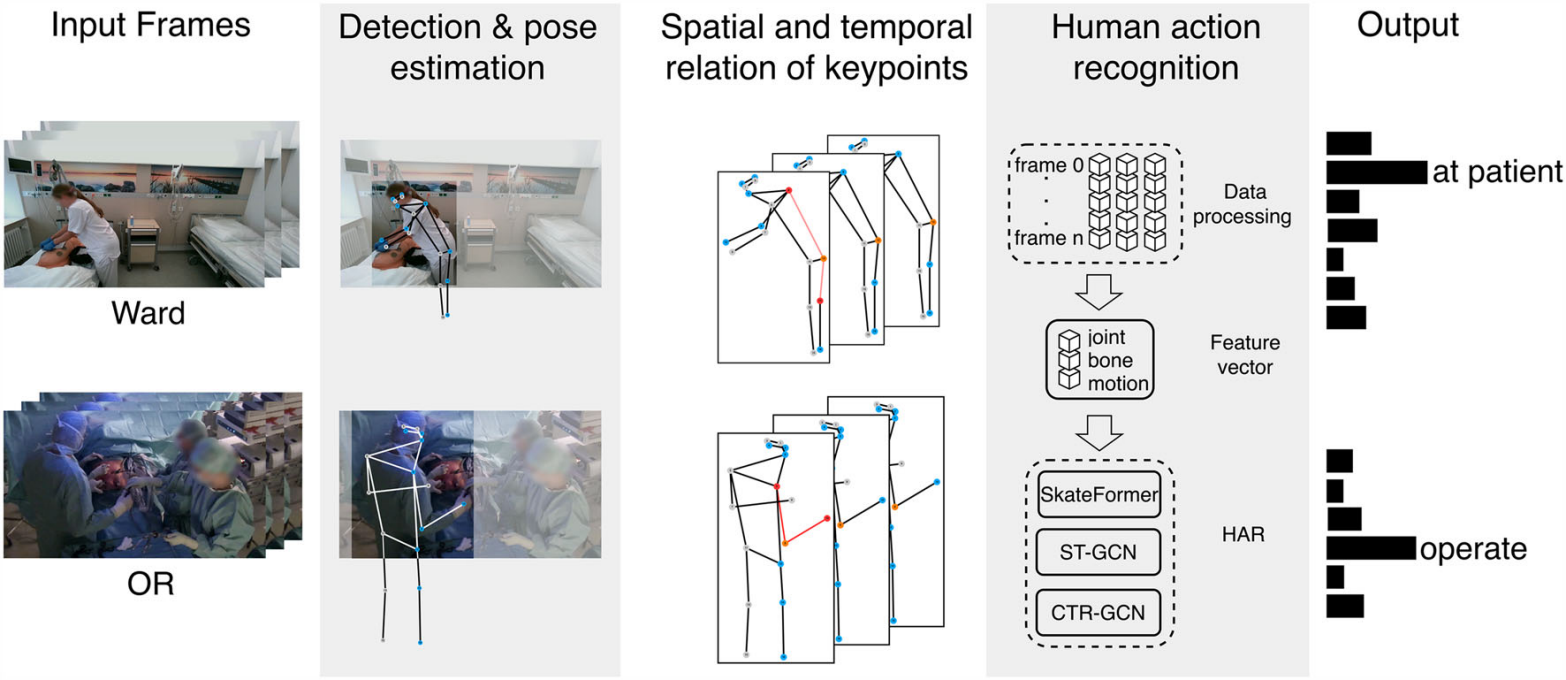
The figure depicts the framework of human action recognition applied to the Ward and OR dataset using human pose recognition. Initially, the person of interest is detected in the input frames. Pose estimation then generates a 2D skeleton for the detected person, with keypoints saved for each frame. The data are subsequently processed into features and edges, encoding joint, bone, and motion characteristics. An action recognition technique is then applied, and the resulting output is seen

To summarize, we introduce human action recognition for medical staff to detect key action classes necessary to introduce robotic assistance in healthcare environments. The conceptual overview of our work is illustrated in Fig. 1. The key contributions are:

- *Novel application*: Introducing HAR in Ward and OR scenarios to address challenges specific to medical environments.
- *Robotic collaboration*: Identifying key medical actions to facilitate effective human–robot interaction without intentional staff input.
- *Method comparison*: Evaluating state-of-the-art pose-based HAR methods on medical action datasets.

## Related work

Leveraging action recognition for coexistence and cooperation with robotic systems has been explored in prior work, such as in an assembly task by Zhang et al. [9]. In this, the pose is used to identify the transition between three action states: installation, handover, and standing. When an individual’s actions are known, the response can be adapted to incorporate context, enabling the implementation of different robot movement strategies. Bergner et al. [10] show an approach for user-centered design of human robot cooperation in an assembly context and focus on creating a work environment that enhances co-working for the human. The application of barriers to identify suitable tasks and design restrictions based on human needs aims to enable more efficient tasks that achieve high levels of usability, acceptance, and perceived safety.

Recent work by Liu et al. [11] used pose recognition in the OR to analyze movement patterns. The use of OR images to gain contextual knowledge has been previously employed by Wagner et al. [12] by fusing multimodal data, including RGB images. The authors show how the instrument can be anticipated through this framework. The ability to anticipate the next instrument is also motivated by the introduction of robotic assistance [3]. In the multi-sensor framework by Zhou et al. [2] next-to-body poses, sensors such as EEG, EMG, and sound for the precision of handover intention delivered a better anticipation of the intent of a handover in an OR mock-up than human observers.

In summary, the current research efforts have focused mostly on the application of human action recognition as a strategy for phase recognition in the OR and do not highlight the individual needs of medical staff. Additionally, the environment of the patient station is not yet permeated by research with robotic systems. Works in line with our contributions have been previously motivated [6].

The following section provides an overview of strategies for human pose estimation based on skeleton data and highlights key reference works. Human action recognition has been approached with a multitude of machine learning techniques. Convolutional neural networks (CNNs) transform spatial skeleton data into pseudo-images for image feature extraction but struggle with temporal connections. Recurrent neural networks (RNNs) model temporal data effectively but require network combinations for optimal skeleton feature incorporation. Graph convolutional networks (GCNs) are popular for combining spatial and temporal data, with static (constant skeleton connections) and dynamic (changing topologies) variations. Transformer-based approaches enhance interconnections between nonphysical joints. [13]

The proposed *spatiotemporal graph convolutional networks (ST-GCN)* by Yan et al. [14] laid the base for many works investigating GCN approaches for HAR. The ST-GCN approach aims to employ the spatial configuration of joints as well as the temporal change. The connection over two dimensions is achieved by linking joints by edges in each frame while also drawing edges between frames.

*Channel-wise topology refinement graph convolution (CTR-GCN)* by Chen et al. [15] does not limit the connections drawn to a generally learned skeleton but adds a channel-wise topology. That means a correlation, including the strength of the connection, is added between links that aren’t connected physically, like the head and hand, based on a dynamic linking.

The *The skeletal-temporal transformer (SkateFormer)* approach by Do et al. [16] groups joints and frames based on the skeletal-temporal relations, namely, the skate-type. Within a partition, a self-attention scheme is utilized. This approach highlights the relationship between joints in the physical space. The temporal relation may need different associations of frames depending on whether the action is local and short term or global, where more distant frames are of interest.

## Methods

## Task formulation

The objective of this work is to recognize the handover actions performed by medical staff in clinical settings, which can be utilized for the control of robotic assistance. Aiming to generalize the objective, two subsets of video data are used, one set from the OR and one set from the patient ward. The task formulation is constrained by the limited availability of suitable data and the definition of handovers as the primary actions of interest. These constraints lead to a simplification of the problem space, which may affect the generalizability of the approach. The datasets depict medical scenes where the staff in focus is caring for, or operating on a patient, while objects are passed to them. Suitable action categories are defined for the classification of interactions while focusing on the receiving party.

**Fig. 2:**
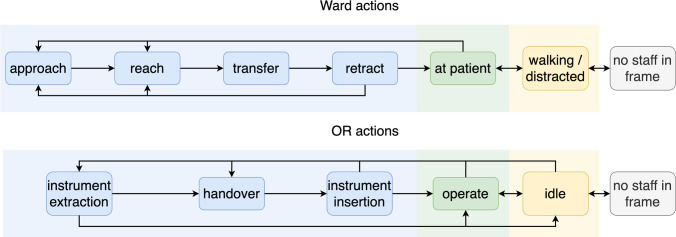
Actions used for the patient and the OR scenario. The Ward actions are split into four handover steps *approach, reach, transfer*, and *retract* accompanied by three surrounding actions, *at patient*, *walking/distracted*, and *no staff in frame*. For the OR, three handover states can be identified, *(instrument) extraction, handover*, and *(instrument) insertion* with three accompanying actions, *operate, idle*, and *no staff in frame*

The extraction of human poses is a prerequisite for further processing but not trivial as it relates to a vast research base itself. To understand and reduce uncertainties introduced by the pose estimators, four algorithms are evaluated by comparing the detection results with the ground truth. The keypoints and confidences of the person of interest are extracted with the library MMpose [17] and saved in a csv format. The acquired data reshaped in weighted multi-stream vectors are then used to apply the HAR strategies, ST-GCN, CTR-GCN, and SkateFormer introduced in the related work. The correct action class is to be determined for the validation data set.

### Relevant actions

With the target of integrating robotic assistance into the given scenarios, a crucial action to recognize is the handover of instruments or medical material. The robotic system, positioned next to the receiving party, acts as an assistant. The handover process after Strabala et al. [18] is split between the physical and social-cognitive processes. The socio-cognitive process establishes the intention of what is handed over, when and where. The physical handover is divided into three steps, approach, reach, and transfer. The carrying or approach is reducing the physical distance usually by walking, while reach describes the arm movement to arrive at the final handover point. The definition of transfer by Strabala et al. [18] includes the retraction movement. To achieve a more detailed breakdown of steps, the finer-grained perspective on object transfer proposed by Ortenzi et al. [19] is utilized for transfer and retraction. The intended task afterward, with the object, should be included to accurately represent the sequence [19].

This leads to six phases that can generally be seen in handovers: *Idle, approach, reach, transfer, retract*, and *use*. The handover phases may vary and should be adopted according to the situation and context [18].

The handover phases in the patient ward can be mapped to the actions found in the literature. Signaling readiness for a handover in the context of minimally invasive surgery may not include approach and reach from the giver’s perspective but the extraction of the instrument out of the trocar and a steady holding position after extraction. Similarly, the end of a handover is not a retraction but can be marked with the insertion of the instrument. The actions we use for the scenarios are illustrated in Fig. 2.

### Dataset

We focus on applying techniques to scenarios commonly encountered in hospitals. Our study utilizes two proprietary datasets, OR and Ward, developed at the University Hospital of the Technical University of Munich. The deliberate use of two distinctly different datasets allows us to address standardized procedures that are well suited for automation. Moreover, the contrasting clinical contexts are expected to improve the applicability and generalizability of the proposed approach. The handover actions in each dataset were annotated according to the phases described previously.

**Fig. 3:**
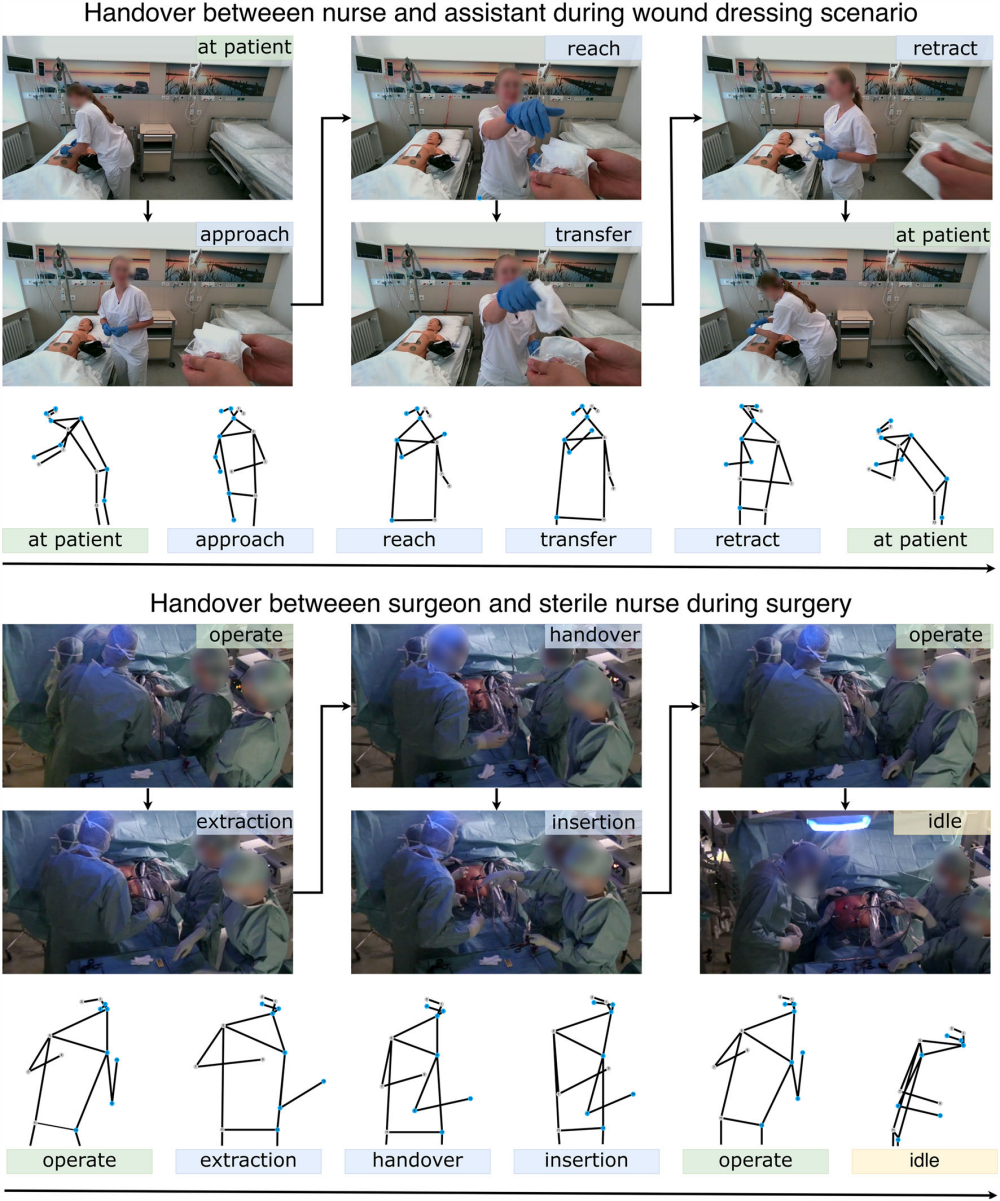
The graph depicts exemplary frames of the Ward and OR dataset, respectively, representing the handover phase. For each frame, the estimated pose is illustrated

The Ward dataset consists of twenty recreated scenes of a wound dressing at a dummy patient with a resolution of $848\,{\times }\,480$ pixels. The dressing changes were recorded at 30 frames per second (fps), performed by ten participants in ward uniforms on a dummy in a patient room, amounting to 31,328 frames in total. To align with medical practice, the participants, TUM University hospital staff, were given written instructions on the appropriate treatment for the injury, which is placed on the dummy to reenact each step of the wound dressing, with added pauses, distractions, and moments of focus before requesting and receiving materials. The scene includes the staff with the dummy patient, while the person handing over items remains outside the visible perimeter, as shown in Fig. 3. The annotation into action classes defined above, as shown in Fig. 2, was done on a frame-by-frame basis.

The OR dataset consists of 14 recorded operations consisting of cholecystectomies and sigmoid resections with a resolution of $1920\,{\times }\,1080$ pixels at 50 fps and amounts to 344,000 frames. The camera footage shows the surgical field and the surrounding staff as shown in Fig. 3. The videos were annotated with the action classes depicted in Fig. 2 at 10 fps by medical experts with the annotation tool CVAT. The dataset comprises videos captured during real surgical procedures, inherently including deviations and anomalies such as unexpected movements.

Table 1 shows the number of frames of the target classes for both datasets. The class imbalance seen by the frame count will be addressed with the evaluation metrics.

**Table 1:** Number of frames of the target classes for both scenarios Ground truth annotations are in COCO-style format from a representative video per dataset

Ward		Operating room	
Action class	Number	Action class	Number
Approach	410	extraction	10,072
Reach	514	handover	18,439
Transfer	753	insertion	14,873
Retract	678	operate	260,622
At patient	26,624	idle	31,799
Walking	1663	no staff	8195
No staff	686		

### Implementation

As visible in the concept in Fig. 1, the implementation consists of the steps detection and pose recognition, keypoint processing, and the application of machine learning models. Different pose detection libraries show a good estimate for the Ward data. The OR dataset proved to be challenging for standard human pose detection, since the light is dimmed, and the subjects wear sterile, wide uni-color gowns that blend in with the background. The MMpose toolbox makes the use and test of various benchmark models available [17].

Pose recognition serves as the foundation for our action recognition. Consequently, it is important to evaluate the accuracy of the extracted skeleton data before employing it as an input stream, particularly when aiming to avoid manual annotation of the entire dataset. For this purpose, four algorithms are evaluated for their suitability in the two use cases. The topdown Swin Transformer [20], with a two-stage architecture, achieves high precision in pose estimation but requires more computational time. The YOLO-Pose algorithm [21] is designed for multi-person pose detection in crowded environments. Additionally, RTMpose3D [22] is looked at, using a topdown approach with CSPNeXt targeting lower computational effort and the additional opportunity to extract 3D pose information for future 3D point estimation. Furthermore, SimCC [23] is selected as it achieves good performance, especially in low-resolution settings. The extracted poses are saved in a csv format with the id, keypoints, confidences, and bounding box. Metrics comparing pose estimates to the ground truth across four algorithms inform the choice of a suitable estimator for each dataset.

The implementation of HAR detection strategies is built on state-of-the-art research with available code bases. For a differentiated view, three techniques: ST-GCN, CTR-GCN, and SkateFormer, are used for action recognition, each developed at different stages of recent advancements in the field. The input frames are used for pose recognition with MMpose, and the resulting pose data with the spatial and temporal relation of keypoints are then fed into the adapted action recognition techniques.

Data augmentation techniques enhance the diversity of training datasets and improve the model’s generalization. The utilized augmentations include scaling, shifting, rotation, and mirroring, each applied with a specified probability.

The features used for the recognition task are separated into three streams: first, the joint features, which are the 2D coordinates in pixel format, second, the bone features defined by a vector between the anatomical connections of the joints, and third, the motion feature, which is the change over time of the position of points between two frames. The three features are put into the model with different weights. The weights are selected after optimization and reported together with the results.

Leveraging the implemented action recognition, fixed-interval action anticipation is applied by shifting the labels by a predefined time (0.5–2 s) [24]. This enables the prediction of future actions. Results for different anticipation horizons are reported.

### Evaluation metrics

The pose estimation algorithms are evaluated by comparing their predicted skeletons to ground truth annotations, using key performance metrics. The ground truth includes a wide range of body poses and levels of occlusion to ensure a comprehensive assessment. We report object keypoint similarity (OKS) and mean average precision (AP), calculated over intersection over union (IoU) thresholds from 0.50 to 0.95, following the COCO-style evaluation protocol. Additionally, we include the percentage of detected joints (PDJ), normalized by head size, at a threshold of 0.1. For further analysis, we examine temporal jitter, calculated as the average variation in joint positions between consecutive frames (in pixels), normalized by the ground truth. We also analyze the cosine similarity between the outputs of different algorithms to assess consistency.

The performance of the three action recognition methods on the Ward and OR dataset is assessed by common metrics of classification tasks like precision, recall, the *F*1 score, and accuracy. The definition of the actions as shown in Fig. 2 leads to an imbalance of available data per action. As the state *at patient* and *operate* are the main activities in the datasets shown in Table 1. For this reason, using weighted-averaged metrics is preferable in order to account for the asymmetry in terms of data and class representation. The weighted average computes the mean for each class and adjusts the results based on the number of instances within each class, ensuring that larger classes have a proportionate influence on the overall average. The weighted average *F*1 scores for anticipation are reported for four temporal offsets $\Delta =[0.5s,1s,1.5s,2s]$. Furthermore, confusion matrices are included for the best-performing model for a visual representation of the predictions. For the Ward dataset, a five-fold cross-validation is used, while a four-fold cross-validation is applied to the OR dataset. Additionally, multi-class receiver operating characteristic (ROC) curves and corresponding area under the curve (AUC) scores are reported for each fold.

**Table 2:** Comparison of pose estimation methods based on PDJ@0.1, OKS, AP over IoU thresholds [0.50 : 0.95], and temporal jitter . Ground truth annotations are in COCO-style format from a representative video per dataset

Method	PDJ	(m) OKS	(m) AP	Jitter
*Ward*				
RTMpose3D [22]	0.6031	0.9126	0.458	4.498
Swin transformer [20]	0.5790	0.8929	0.449	4.669
YOLO-Pose [21]	0.5513	0.8758	0.432	4.473
SimCC [23]	0.4894	0.8335	0.413	5.759
*Operating room*				
RTMpose3D [22]	0.4689	0.5419	0.099	40.975
Swin transformer [20]	0.4784	0.5331	0.101	31.446
YOLO-Pose [21]	0.3527	0.4345	0.043	128.860
SimCC [23]	0.4060	0.4923	0.054	28.100

## Results

The proposed skeleton-based action recognition aims to identify actions in medical scenarios that could enable robotic assistance in operating rooms and wards for handover tasks of surgical instruments or medical equipment. First, we report the PDJ, OKS, AP, and jitter of four pose estimators on the Ward and OR data for algorithm selection. Building on the extracted skeleton, we focus on analyzing three implemented action recognition techniques. Multi-stream features, joint, bone, and motion are fed into the models, to be combined afterward with an optimized weight for each feature. Results of the three models with their weighted-averaged metrics can be seen in Table 3.

**Table 3:** Weighted-averaged metrics for the task of skeleton-based action recognition The performance of different methods for both theWard and OR scenarios is compared. The averaged metrics are reported (dataset and five-fold for the Ward dataset

Method	(wm)AP	(wm) AR	(wm) A*F*1	(wm) AA
*Ward*				
ST-GCN [14]	$0.939 \pm 0.010$	$0.931 \pm 0.017$	$0.933 \pm 0.014$	$0.931 \pm 0.017$
CTR-GCN [15]	$0.940\pm 0.007$	$0.930 \pm 0.013$	$0.933 \pm 0.011$	$0.930 \pm 0.013$
SkateFormer [16]	$0.949 \pm 0.006$	$0.938 \pm 0.011$	$0.941 \pm 0.009$	$0.938 \pm 0.011$
*Operating room*				
ST-GCN [14]	$0.782 \pm 0.041$	$0.709 \pm 0.053$	$0.736 \pm 0.045$	$0.709 \pm 0.053$
CTR-GCN [15]	$0.758 \pm 0.033$	$0.663 \pm 0.089$	$0.695\pm 0.062$	$0.663 \pm 0.089$
SkateFormer [16]	$0.734 \pm 0.055$	$0.755\pm 0.061$	$0.728 \pm 0.040$	$0.755 \pm 0.061$

### Pose estimators

Table 2 presents a comparison of the pose estimation results obtained from four algorithms, Swin Transformer, RTMpose3D, SimCC, and YOLO-Pose. For the Ward data, the PDJ with an error threshold of 0.1 is highest for RTMpose3D, with a value of 0.6031, indicating strong localization of keypoints. The model also achieves an OKS of 0.9126, which reflects excellent spatial alignment when accounting for scale and visibility related accuracy. An AP of 0.458 further shows high confidence in correctly aligned keypoints. All models show similar jitter between frames, with RTMpose3D recording a value of 4.498 pixels per frame. This suggests comparable temporal stability across models and no clear advantage in motion consistency.

Swin Transformer and RTMpose3D achieved similarly low scores on the OR data, indicating increased difficulty in pose estimation under this scenario. Swin Transformer had slightly better results, as indicated by a PDJ@0.1 of 0.4784, and AP of 0.101, with a lower OKS of 0.5331. The low AP on the OR data reflects the low confidence of the model, although keypoints are not deviating significantly from the ground truth. A higher jitter of 40.975 pixels per frame observed for RTMpose3D may indicate less stable keypoint tracking and a poorer representation of motion over time.

Nevertheless, the cosine similarity between all model predictions for the ward scenario exceeds 0.99, indicating a very high degree of similarity while staying above 0.98 for the OR, except YOLO-Pose with 0.97. This high cosine similarity suggests that the extracted pose structure and shape remain consistent across most models and are unlikely to significantly affect action recognition, as the global position is not as relevant.

**Table 4:** Weighted-average *F*1 scores for action anticipation

Time interval $\Delta$	0.5 s	1 s	1.5 s	2 s
*Ward*				
ST-GCN [14]	$0.9128 \pm 0.018$	$0.898 \pm 0.018$	$0.891\pm 0.019$	$0.878 \pm 0.014$
CTR-GCN [15]	$0.914 \pm 0.013$	$0.904 \pm 0.019$	$0.895 \pm 0.016$	$0.878 \pm 0.018$
SkateFormer [16]	$0.932 \pm 0.013$	$0.923 \pm 0.011$	$0.910 \pm 0.013$	$0.911 \pm 0.015$
*Operating room*				
ST-GCN [14]	$0.726 \pm 0.031$	$0.712 \pm 0.022$	$0.701 \pm 0.022$	$0.692 \pm 0.038$
CTR-GCN [15]	$0.696 \pm 0.060$	$0.683 \pm 0.057$	$0.681 \pm 0.0514$	$0.623 \pm 0.112$
SkateFormer [16]	$0.722 \pm 0.043$	$0.729 \pm 0.040$	$0.716 \pm 0.039$	$0.720 \pm 0.031$

Performance is compared across varying anticipation intervals for different methods in both the Ward and OR scenarios. The average *F*1 scores (in $\%$) along with standard deviations (±) are presented, based on four-fold cross-validation for the OR dataset and five-fold cross-validation for the Ward dataset

**Fig. 4:**
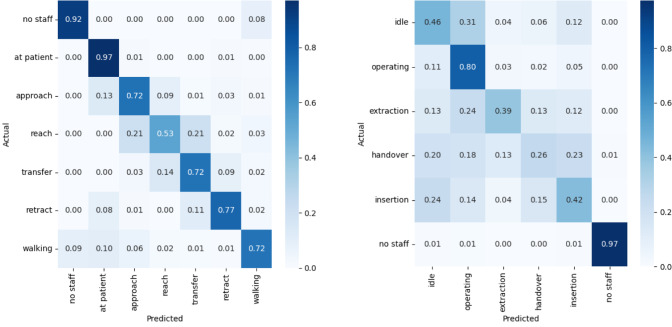
Multi-class confusion matrices for both scenarios and best model. Each cell shows the row-wise normalized proportion of predictions, with values ranging from 0 to 1, showing the recall for each action class a Confusion matrix for Ward scenario. Model: SkateFormer. Streams: joint, bone, motion 0.5, 0.2,0.3. b Confusion matrix for OR scenario. Model: ST-GCN. Streams: joint, bone, motion 0.35, 0.3, 0.35

For the OR, the topdown heatmap with Swin Transformers [20] trained on Coco showed a good performance and is used. For the Ward scenario, the pose recognition is built on RTMpose3D [22] choosing the benefit of 3D pose estimation over similar results of the Swin Transformer.

### Ward scenario

**Fig. 5:**
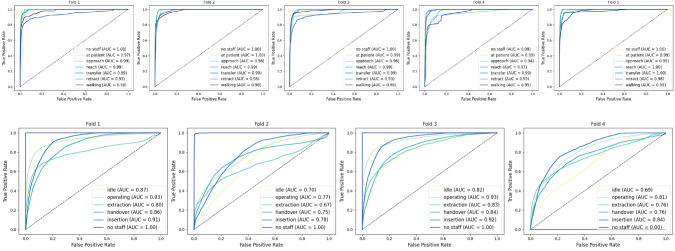
Receiver operating characteristic curves and corresponding area under the curve metrics for the SkateFormer model in a multi-class classification setting. Each curve represents the model’s performance on one class versus the rest, providing insight into class-wise discrimination capability . a ROC curves for the Ward action recognition for each of the five folds. b ROC curves for the OR action recognition for each of the four folds

ST-GCN with a feature weight of $\{0.3, 0.5, 0.2\}$ and CTR-GCN with a weighting vector of $\{0.35, 0.35, 0.4\}$ show a lower *F*1 score than SkateFormer with the multi-stream weight of $\{0.5, 0.2, 0.3\}$ displayed in Table 3. Particularly, the handover steps of approach and reach are proven to be hard to distinguish. The *F*1 score for reach for SkateFormer is $0.575 \pm 0.038$, for CTR-GCN $0.539 \pm 0.090$ and for ST-GCN $0.528 \pm 0.028$. Interestingly, while SkateFormer and CTR-GCN have the worst *F*1 score by class, for reach ST-GCN has the lowest *F*1 score for approach with $0.481 \pm 0.079$. Figure 4a depicts the confusion matrix for the ward scenario with the best-performing model, SkateFormer. Although the prediction of the approach is improved compared to other models, reach is still confused with approach and transfer. This can be due to either the similarity in movement or the lack of concise movement by the untrained participants. As the filmed individuals received instructions, sometimes reach was performed with hesitation or with retracting in between because of the lack of knowledge about the situation. Although ST-GCN and CTR-GCN appear to have nearly the same weighted average *F*1 score, they show a difference in the class representation sensitive, macro-weighted *F*1 score with CTR-GCN achieving $0.701 \pm 0.038$ and ST-GCN achieving $0.695 \pm 0.037$. Figure 5a illustrates the model’s class-separation performance using multi-class ROC curves and the corresponding AUC scores. Despite the impact of limited class representation on detection accuracy, the model maintains a low false positive rate. The lowest reported AUC is 0.93, indicating strong discriminative capability across all classes.

Transferring these results to anticipation with fixed time interval shifts shows similar average weighted *F*1 scores, as presented in Table 4. Nevertheless, anticipation performance for minority classes, such as transfer at $\Delta = 1s$, is substantially lower than in the recognition task, with an *F*1 score of $0.591 \pm 0.089$ compared to the recognition result of $0.717 \pm 0.056$ both with SkateFormer. Most noticeable is that the start of the handover, the approach, is difficult to differentiate from other classes with a recognition *F*1 of $0.522 \pm 0.098$ and an anticipation *F*1 at $\Delta =1s$ of $0.350 \pm 0.084$ with SkateFormer.

### OR scenario

ST-GCN with a feature weight of $\{0.35, 0.3, 0.35\}$ reaches the highest *F*1 score compared to SkateFormer with a weight vector of $\{0.5, 0.2, 0.2\}$ and CTR-GCN with weights of $\{0.4, 0.25, 0.35\}$ as shown in Table 3. The OR confusion matrix with ST-GCN shown in Fig. 4b visualizes the difficulty of separating the handover classes. The three actions comprising the handover phase are conflated with operating, as the action classes of a handover reflect only a small portion of the process in the OR, leading to the accumulation of actions not central to our focus in either the operating or idle class. While simplifying the annotation and task formulation, this led to worse detection results. The macro-weighted *F*1 score for ST-GCN of $0.470 \pm 0.089$ highlights the detection issues by class. Examining the multi-class ROC curves in Fig. 5 shows noticeable differences in classification performance between folds, which could point to inconsistencies in the data or sampling variations. In fold two, the model shows lower performance for the class extraction, with an AUC of 0.67. In fold four, the no staff class shows inverted results, due to the absence of this label in the validation set.

Similarly to the Ward, the time shift average weighted *F*1 scores, visible in Table 4, show no distinct difference, but affects the minority classes. Handover is anticipated at a $\Delta = 1s$ with an *F*1 of $0.221\pm 0.052$ compared to the recognition *F*1 of $0.280 \pm 0.053$ with the ST-GCN model.

We report the partially successful application of HAR techniques on both datasets. Summing up, SkateFormer shows by a small difference the best results for recognizing the actions in the Ward and ST-GCN in the OR dataset as shown in Fig. 3. The Ward dataset showed a persistently higher accuracy for each implemented model, and this could be due to more distinguishable movements in the patient ward setting. The OR actions depict very slight movements in the handover as shown in Fig. 3, which may have contributed to the lack of accuracy in separating the classes.

Anticipation with a time shift of 1 s does still perform similarly in terms of *F*1 scores. The feature vector optimization indicates that bone and motion features become more important compared to joints relevant for anticipation.

## Discussion

Our work provides an initial exploration of human action recognition and anticipation in healthcare scenarios. The actions used as detection targets are short sequences in time, which increases the challenge of associating the recorded data with the target movements. Actions such as turning to the assistant for visual confirmation of their process speed are added to the majority class, operating, while it might have been suited for a new class for precise detection results. Nevertheless, the action recognition results show a good performance with SkateFormer and ST-GCN, especially with the re-enacted data of the Ward. The two datasets differ in subject, context and conditions. The results shown in Table 3 show the ability to detect the defined action classes shown in Fig. 2 regardless of these differences. Anticipation remains challenging, although classes are learned and show a moderate *F*1 score, and minority classes have low anticipation results. Sliding-window prediction techniques may help manage inconsistent results within the proposed model structure. A handover can be modeled as a transition between discrete states, similar to a state machine, and can therefore be framed as the prediction of the next likely step. In robotic applications, anticipating key human actions such as approaching or reaching is essential. Although false positives may cause the robot to initiate a handover prematurely, the system can adapt its plan when confidence remains low, supporting flexible interactions.

The vision of humans and technical systems working hand in hand fluently has been motivated in research [2, 6, 12]. The need for action recognition systems is apparent in the context of process optimization, decision support systems, and the integration of robotic systems. The detection task of medical handovers will become a key milestone for context-aware operating room or patient ward scenarios. With the presented results, we hope to motivate further exploration of skeleton-based action recognition in medical scenarios.

An apparent limitation of the Ward dataset is the use of a mock-up scenario with clinic staff instead of filming an actual wound dressing. During the mock-up procedure, some participants hesitated in their actions as they were following written instructions, which may have blurred the lines of what action is currently done. Future research should incorporate dataset collection during patient care. Through our work, we aim to encourage systematic data collection in this underexplored area. In particular, incorporating datasets related to care procedures like drainage replacement and urinary catheter removal would provide valuable complementary data.

While there are currently no publicly available datasets that provide the detailed, interaction-specific annotations needed to study handover scenarios, and existing datasets from the operating room domain could still be useful in future work. These datasets may be fully re-annotated with help from medical experts to include the level of detail required for analyzing handovers. The OR dataset consists of cholecystectomies and sigmoid resections, which are standardized procedures and, usually, a less volatile detection target for recognizing action patterns. Nevertheless, real-world data are always more prone to variability and demands a closer look into the definition and annotation of actions. Particularly in medical scenarios, the data availability is limited. Therefore, we believe that an abstraction into keypoints may support the generalization with limited datasets. For this reason, although better and more data will provide improved detection results, this should not be the first step of action to refine our work.

To conduct the first validation step toward generalizing our framework, we applied it to another surgical domain. For this, we utilized the MM-OR dataset [25], which has multi-camera views of total and partial knee arthroplasties. Although MM-OR represents a controlled, simulated environment, similar to our Ward dataset, it provides an alternative context by focusing on a different procedure, like the partial knee replacement, and can be included in the same pipeline by featuring static camera angles. Given the resource-intensive nature of annotating complex surgical video data, we adopted a sparse annotation strategy to assess the model transferability. We manually annotated three sequences, capturing over 20 handover events. This targeted ground truth enabled an exploratory assessment of the performance of the OR model, trained exclusively on our primary dataset, when applied to the novel MM-OR sequences for action recognition. We therefore deployed the best-performing model from the OR dataset experiments. Evaluation on the MM-OR dataset achieved a weighted average *F*1 score of 0.658. Considering that no fine-tuning or transfer learning was applied, this result underscores the model’s inherent generalization capability and demonstrates the feasibility of direct deployment across distinct surgical domains.

For both datasets, the filmed scenes and, therefore, the action classes are of the scenario with a human assistant. This leads to movements from the medical staff in focus that may not occur in a scenario with a robotic system. Retrieving information to be used for robotic assistance by analyzing movements without these technologies will lead to limitations in transferability.

Furthermore, the data were collected with a static camera. This setup does not allow for occlusions or the shift of the scene to another area. A possible solution for the OR would be a multi-camera setup and fusion of information, while in the more variable patient ward, a robot equipped with a camera could actively follow the movements of the person of interest. As the temporal aspects and interaction speed vs. delays might play an important role, motion tracking and videography might be considered in follow-up studies.

The shown HAR could be adapted to more interactions than the presented handover. The chosen camera angle is advantageous for perceiving the handover, especially in the Ward dataset. Other actions, like the application of wound dressing to the dummy patient, were not as visible and are not able to be represented precisely with action classes. Similarly, in the OR laparoscopic procedures can be best described by the analysis of intraabdominal video footage. To represent the spectrum of different actions, a multimodal view would be necessary.

We motivate this work with the foresight of automation, but are aware of the clear limitations that a mere action recognition and fixed-interval anticipation opens the solution sphere to a larger implementation pipeline. To support human–robot interaction strategies, anticipation has to give a reliable result in a reasonable time frame to determine the robot’s action.

## Conclusion

The aim of the shown action recognition is the extraction of spatiotemporal information for the future application of robotic systems into medical environments. The need for effective teamwork with robotic systems motivates the identification of the key collaborative actions in the operating room as well as in the patient ward. We could successfully present an *F*1 score of $0.941 \pm 0.009$ for the Ward dataset with the best-performing model SkateFormer and an *F*1 score of $0.736 \pm 0.009$ for the OR dataset with ST-GCN. We will focus on the detection improvement, especially for the OR classes. Future work will focus on the use of the actions for medical support systems in multimodal systems and the application of the framework for robotic assistance in handover situations.

## Data Availability

The data supporting the presented work are available from the corresponding author upon request. Due to privacy restrictions, the datasets are not publicly accessible but are available upon request to qualified researchers, subject to appropriate agreements and institutional oversight.

## Declarations

### Code availability

The underlying code for this study is available at https://github.com/ResearchgroupMITI/human-action-recognition.
